# Peroxisome Deficiency Dysregulates Fatty Acid Oxidization and Exacerbates Lipotoxicity in *β* Cells

**DOI:** 10.1155/2021/7726058

**Published:** 2021-08-22

**Authors:** Hongbo Guan, Yanyan Guo, Liangliang Zhu, Yisheng Jiao, Xiaomei Liu

**Affiliations:** Key Laboratory of Maternal-Fetal Medicine of Liaoning Province, Department of Obstetrics and Gynecology, Shengjing Hospital of China Medical University, Shenyang 110004, China

## Abstract

An adverse intrauterine environment impairs the development of pancreatic islets in the fetus and leads to insufficient *β* cell mass and *β* cell dysfunction. We previously reported that Pex14, a peroxin protein involved in the biogenesis and degradation of peroxisomes, is markedly reduced in the pancreas of an intrauterine growth restriction fetus and last into adulthood. Peroxisomes function in a wide range of metabolic processes including fatty acid oxidization, ROS detoxification, and anti-inflammatory responses. To elucidate the impact of downregulation of the Pex14 gene on *β* cell, Pex14 was knocked down by siRNA in INS-1 cells. Pex14 knockdown disturbed peroxisomal biogenesis and dysregulated fatty acid metabolism and lipid storage capability, thereby increased ROS level and blunted insulin secretion. Moreover, Pex14 knockdown upregulated inflammation factors and regulators of endoplasmic reticulum stress. The lipotoxicity of fatty acid (including palmitic acid and linoleic acid) in *β* cells was exacerbated by knockdown of Pex14, as indicated by H_2_O_2_ accumulation and increased programmed cell death. The present results demonstrate the vital role of Pex14 in maintaining normal peroxisome function and *β* cell viability and highlight the importance of a functional peroxisomal metabolism for the detoxification of excess FAs in *β* cells.

## 1. Introduction

The peroxisome is an organelle with multiple biological functions that is widely present in mammalian cells. Human peroxisomes contain more than 80 proteins that can be divided into two categories. The first is constitutive proteins, called peroxins (PEX), which interact with each other and participate in peroxisome biogenesis, division, and proliferation. The second class comprises metabolic, oxidative, and antioxidant enzymes (around 50 in mammals), which are critical for the maintenance of cellular homeostasis and activity [1, 2]. Peroxisomes are involved in a wide variety of metabolic pathways such as *β*-oxidization of very-long-chain fatty acids (VLCFAs), *α*-oxidization and *β*-oxidization of long branched-chain fatty acids (FAs), phospholipid synthesis, reactive oxygen species (ROS) metabolism, and anti-inflammatory functions [3–5]. PEX gene mutations cause defects in peroxisome production and loss of mature peroxisomes, leading to a group of diseases known as peroxisomal biogenesis disease [2]. The underlying pathological mechanism involves impaired oxidization of VLCFAs, which accumulate in the cytoplasm, affecting cell function and embryo development [6, 7]. In addition, the peroxisome can produce ROS and reactive nitrogen species (RNS), as well as remove ROS/RNS. The imbalance between the two processes causes oxidative stress (OS) [8].

Type 2 diabetes (T2D) is a multifactorial disease caused by genetic and environmental factors. Dysfunction of *β* cells is one of the main processes involved in the pathogenesis of T2D [9]. One important mechanism underlying *β* cell dysfunction is glucose and FFA toxic action-induced OS. OS results in glucose intolerance, inhibits insulin secretion and activity, and promotes the onset of T2D [10, 11]. Epidemiological studies show that intrauterine growth restriction (IUGR) increases the susceptibility to adult T2D, owing to the maldevelopment of islets and *β* cell dysfunction [12, 13]. In a previous study, we used a rat model to demonstrate the aberrant expression of pancreatic peroxisome factors in the IUGR fetus induced by malnutrition in utero [14]. Pex14, a peroxisome marker protein involved in the biogenesis and degradation of peroxisomes [15], was markedly reduced in the fetal pancreas that persist into adulthood. Another downregulated protein was Pex3, an integral membrane protein involved in peroxisome biogenesis by interacting directly with Pex16 and 19 that contributes to peroxisome membrane protein (PMP) protein import [16]. Acyl-CoA oxidase (Acox) 1 and peroxisomal 17*β*-hydroxysteroid dehydrogenase type 4 (Hsd17b4), which are rate-limiting enzymes in FA *β*-oxidation (FAO), were downregulated, and this was accompanied by activation of OS in the IUGR fetus. Therefore, we hypothesized that downregulation of Pex14 impairs peroxisome biogenesis and function, leading to *β* cell dysfunction.

In this study, using siRNA to knock down the expression of Pex14, we found that downregulation of Pex14 induced *β* cell death and damaged insulin secretion function by affecting multiple proteins involved in peroxisome biogenesis, FAO, and ROS scavenging, subsequently leading to cell autophagy and apoptosis. These results suggest that the downregulation of Pex14, at least partly, lead to pancreatic dysplasia in IUGR offspring.

## 2. Materials and Methods

### 2.1. Cell Culture and Transfection

The rat INS-1 cell line was purchased from the Type Culture Collection Center of Chinese Academy of Science (Shanghai, China). Cells were cultured in 1640 medium containing 10% fetal bovine serum and 100 U/ml penicillin-streptomycin and maintained at 37°C under 5% CO_2_. The sequences of siRNAs targeting rat Pex14 and a negative control (NC) are shown in Table S1. siRNAs were transfected into INS-1 cells using the siRNA transfection reagent INTERFERin (Polyplus 409-10), according to the manufacturer's manual. At 24 h after transfection, the cells were incubated with the saturated FA palmitic acid (PA) or the unsaturated FA linoleic acid (LA) at concentrations of 30 *μ*M and 10 *μ*M, respectively, for 24 hours. The cell media were then changed to basal media, and cells were collected for subsequent experiments.

### 2.2. RNA Isolation and q-PCR

Total RNA was extracted using the TRIzol reagent (Invitrogen) according to the manufacturer's protocol. cDNA was obtained with the Reverse Transcription System (Takara, Dalian, China). q-PCR was performed using a SYBR Green PCR Kit (Vazyme, Q711-02, Nanjing, China) on a 7500 Fast system (Applied Biosystems). Data were analyzed using the relative quantification (2^–*ΔΔ*Ct^) method with *β*-actin as the endogenous control. Primer sequences are listed in Table S1.

### 2.3. Western Blot Analysis

INS-1 cells were lysed in SDT buffer (4% sodium dodecyl sulfate (SDS), 1 mM dithiothreitol, 150 mM Tris-HCl, and pH 8.0), and protein content was quantified with the BCA method. Equal amounts of protein were separated by 12% (*w*/*v*) SDS–PAGE and transferred to a polyvinylidene difluoride membrane. The membrane was cut into several strips, blocked in TBS-Tween-20 containing 5% (*w*/*v*) skimmed milk for 2 h at room temperature, and incubated with the indicated primary antibodies (Table S2) overnight at 4°C. The membrane was then incubated with secondary antibodies and visualized using a commercial enhanced chemiluminescence detection kit (Millipore, USA). Gel-Pro Analyzer software was used for quantification of immunoblots.

### 2.4. Apoptosis Analysis

After siRNA transfection, cells were collected for apoptosis assays with an Annexin V-FITC Apoptosis Kit (Vazyme, Nanjing, China). Cells were washed with PBS and resuspended in 500 *μ*L of binding buffer. Subsequently, 5 *μ*L of Annexin V-FITC and 5 *μ*L of propidium iodide (PI) were added into the cell suspension and incubated at room temperature for 15 min in the dark. The apoptosis rate of cells in each group was measured using a BD FACSCalibur flow cytometer.

### 2.5. Cell Counting Kit-8 Assay

Cell viability was measured using the Cell Counting kit-8 (CCK8) assay. Briefly, INS-1 cells (5 × 10^3^ cells/well) were seeded into 96-well plates. At 24 h after transfection, cells were incubated in 10% CCK8 solution (Vazyme) at 37°C for 2 h until visual color conversion occurred. The absorbance at 450 nm was measured using a microplate spectrophotometer (BioTek Instruments, Winooski, VT, USA).

### 2.6. Autophagy Detection

Autophagic flux was analyzed using the Cyto-ID® Autophagy Detection Kit (Enzo Life Sciences, ENZ-51031-K200) in INS-1 cells. Briefly, after treatment with LA or PA for 2 h, cells (1 × 10^6^ cells/mL) were incubated with Cyto-ID Green Detection Reagent (1 *μ*L/mL) adding in culture medium for 30 min at 37°C and 5% CO_2_ in the dark. Images were taken with a Nikon inverted microscope at 200x amplification.

### 2.7. Cellular ROS Detection

Intracellular ROS were detected with fluorescent probes 5-(and-6)-chloromethyl-2-,7-dichlorofluorescin diacetate (DCFH-DA, indicator of H_2_O_2_). INS-1 cells were harvested and incubated DCFH-DA (10 *μ*M, 30 min) at 37°C in the dark, according to the manufacturer's protocol. For fluorescence quantitative analysis, the cells were collected and analyzed by flow cytometry. At least 10,000 events were analyzed.

### 2.8. Immunofluorescence (IF) and Nile Red Staining

INS-1 cells were fixed for 15 min with ice-cold 4.0% paraformaldehyde and then permeabilized with 0.2% Triton X-100 for 30 min. IF staining was performed by incubating cells with the indicated primary antibodies (Table S2) at 4°C overnight, and then labeled with fluorescence conjugated anti-rabbit or anti-mouse IgG (Invitrogen, A11029 and A11036, respectively) at 37°C for 60 min, followed by nuclear staining with DAPI. Images were captured with a laser scanning confocal microscopy at the same magnification, ×400. Intracellular lipid droplets were observed with Nile Red staining (0.1 *μ*mol/mL). Images were acquired with the confocal microscopy with magnification of 200x.

### 2.9. Glucose-Stimulated Insulin Secretion (GSIS) Experiment

INS-1 cells were incubated in Krebs-Ringer buffer (KRB, 4.7 mM KCl, 115 mM NaCl, 1.2 mM MgSO_4_, 0.5 mM NaH_2_PO_4_, 1.5 mM CaCl_2_, 2.5 mM NaHCO_3_, 0.1% BSA, and pH 7.4) containing 2.5 mmol/L glucose (basal secretion) or KRB buffer containing 20 mmol/L glucose (stimulatory) for 1 h. An aliquot of the medium was collected for assay of insulin with an ELISA kit (CUSABIO, China) according to the manufacturer's protocol.

### 2.10. Statistical Analysis

Results are presented as the mean ± standard deviation of the mean (SD) from at least three separate experiments. Differences between two groups were analyzed with the unpaired Student two-tailed *t*-test, and one-way ANOVA was used for comparison of more than two groups. *p* < 0.05 was considered statistically significant.

## 3. Results

### 3.1. Pex14 Knockdown Alters Peroxin Expression and Induces Peroxisomal Deficiency in INS-1 Cells

**Figure 1:**
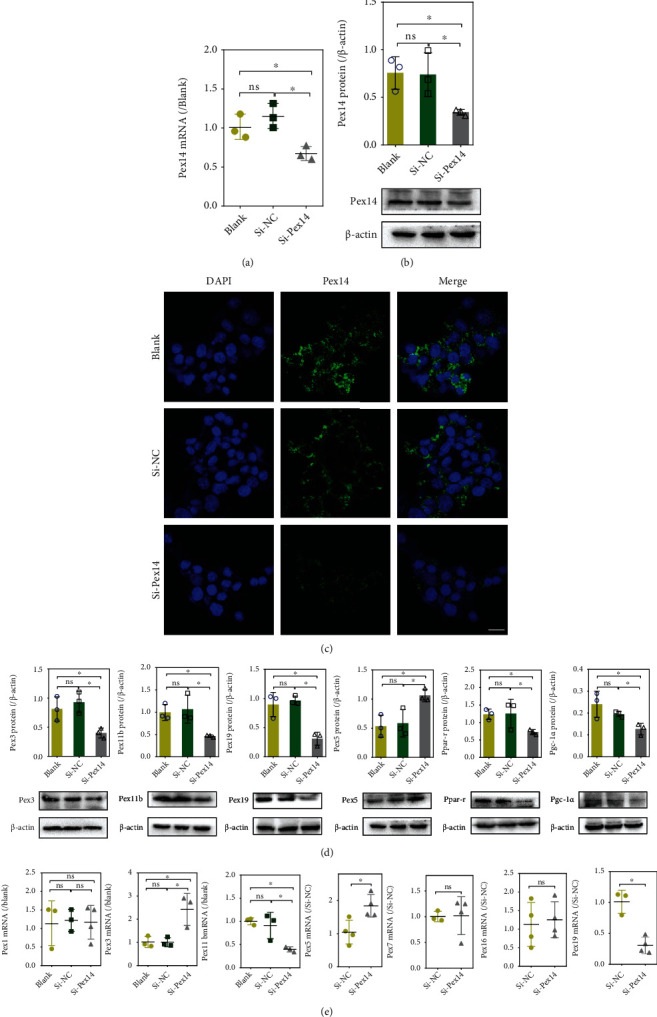
Knockdown (KD) of Pex14 causes defective expression of peroxins implicated in peroxisome biogenesis and degradation. (a, b) Knockdown efficiency of INS-1 cells transfected with si-Pex14 or si-NC was examined by q-PCR (a) and western blotting (b). (c) Representative IF photomicrographs of Pex14 in INS-1 cells (×800 magnification, scale bar 100 *μ*m). (d, e) The expression of peroxins and the transcription factors that regulate peroxins was detected by western blot (d) or q-PCR (e). *n* = 3-4; error bars indicate SD. ^∗^*p* < 0.05, vs. the blank or si-NC group.

INS-1 cells transfected with siRNA against Pex14 showed up to 55% reduction of Pex14 expression compared with scrambled siRNA-transfected cells (Figures 1(a) and 1(b)**)**. The observation that si-NC did not decrease Pex14 expression confirmed that the silencing was specific. IF staining also confirmed that Pex14 were downregulated in si-Pex14 INS-1 cells (Figure 1(c)). Pex14 KD significantly downregulated the protein expression of Pex3, Pex5, Pex19, and Pex11b (Figure 1(d)). q-PCR assays revealed a decrease in the mRNA level of Pex11b and 19, consistent with the protein data. The mRNA level of Pex1, Pex7, and Pex16 were not affected. In contrast, the mRNA level of Pex3 and Pex5 was upregulated by Pex14 KD which was inconsistent with the protein data (Figure 1(e)). We speculate that the increased mRNA expression might be a feedback response to the decreased protein amount. It is interested to find that the transcription factors that regulate peroxisome proliferation, Ppar-*γ* and its coactivator Pgc-1*α*, were both diminished after Pex14 KD (Figure 1(d)).

### 3.2. Fatty Acid Metabolism in Pex14 KD Cells

**Figure 2:**
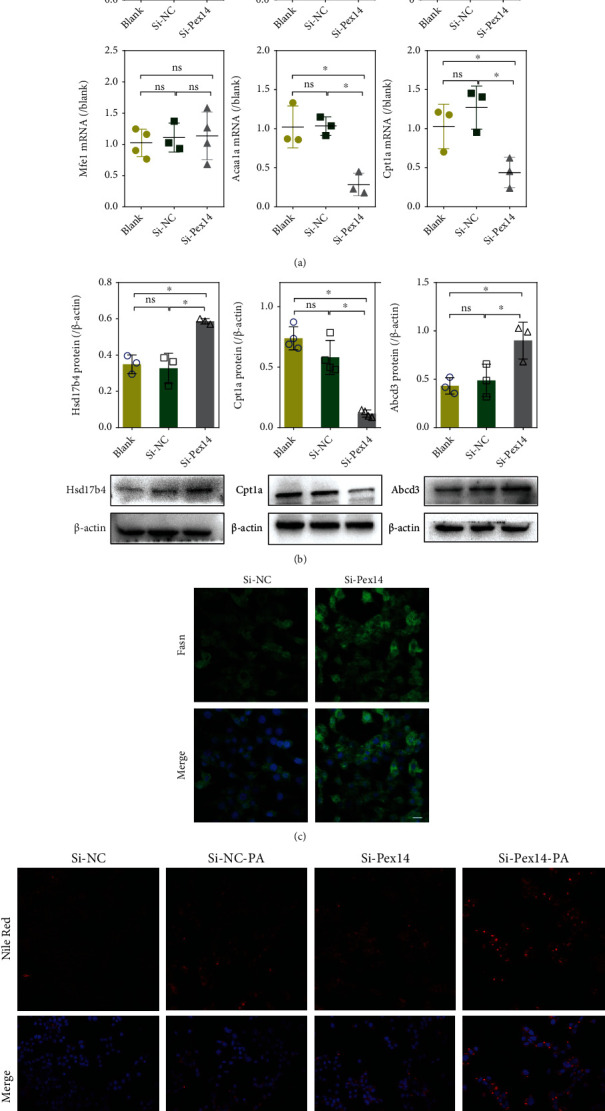
Dysregulated expression of genes implicated in fatty acid metabolism are shown in Pex14 KD INS-1 cells. (a, b) The expression of FAO enzymes and Abcd3 was detected by q-PCR (a) and western blot analysis (b). ^∗^*p* < 0.05, vs. the blank or si-NC group. (c) Fasn protein expression was detected by IF staining (×400), scale bar 50 *μ*m. (d) Nile Red staining of INS-1 cells (×200). Scale bar 100 *μ*m.

q-PCR assay showed that Pex14 KD lead to different variation tendencies in the mRNA level of peroxisome FAO enzymes. Regarding Acox isozymes, Acox1 was downregulated and Acox3 was upregulated, whereas the heterodimer Acox2 was unaffected. The mRNA level of Mfe1 was also unchanged, while 3-ketoacyl-CoA thiolase A (Acaa1a) was decreased (Figure 2(a)). Western blot analysis showed that the protein expression of Hsd17b4 and Abcd3 *was upregulated*, while carnitine palmitoyltransferase-1A (Cpt1a), was decreased in Pex14 KD cells (Figure 2(b)). IF staining demonstrated the enhanced expression of Fasn(Figure 2(c)). Nile Red staining showed that Pex14 KD caused a slight increase in lipid droplets in INS-1 cells compared with the si-NC group. After exposure to PA, Pex14 KD led to much higher predisposition to fat accumulation compared with the si-NC group (Figure 2(d)).

### 3.3. ROS Detoxification and Proinflammatory Factors in Pex14 KD Cells

**Figure 3:**
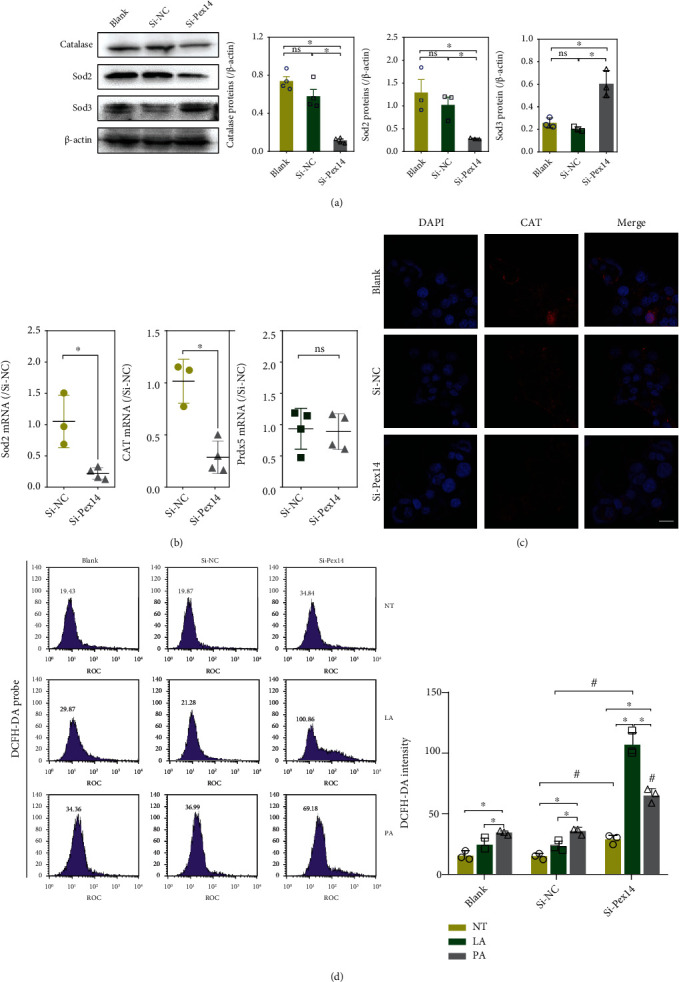
Knockdown of Pex14 aggravated fatty acid-induced intracellular ROS accumulation. (a, b) Antioxidant factors were detected by immunoblot assay (a) and q-PCR (b); ^∗^*p* < 0.05, vs. the blank or si-NC group. (c) IF showed the decreased expression and location of catalase. (d) Intracellular ROS were detected by flow cytometry in INS-1 cells treated with LA or PA using the probes DCFH-DA and the average *G*-mean values were calculated. NT: no treatment. ^∗^*p* < 0.05, vs. the NT or LA group. ^#^*p* < 0.05, vs. the si-NC with or without LA/PA group.

Western blot analysis showed a decrease in the protein expression of antioxidant catalase (CAT) and Sod2 and an increase in Sod3 protein level. q-PCR assay also demonstrated a decrease in mRNA level of Sod2 and CAT. Prdx5, an antioxidant localized in peroxisomes and mitochondria, was unchanged in the mRNA level (Figures 3(a)–3(c)). We further investigated ROS levels in Pex14 KD cells with DCFH-DA. As shown in Figure 3(d), Pex14 silencing caused a marginal increase in H_2_O_2_ in INS-1 cells. Moreover, H_2_O_2_ was markedly increased upon exposure to LA in Pex14 KD cells, whereas LA exposure showed no significant effect on H_2_O_2_ in the blank and si-NC cells. Exposure to PA led to a moderate increase in ROS in blank cells compared with the LA exposure groups, whereas in Pex14 KD cells, PA exposure caused a less increase in ROS levels than LA exposure.

**Figure 4:**
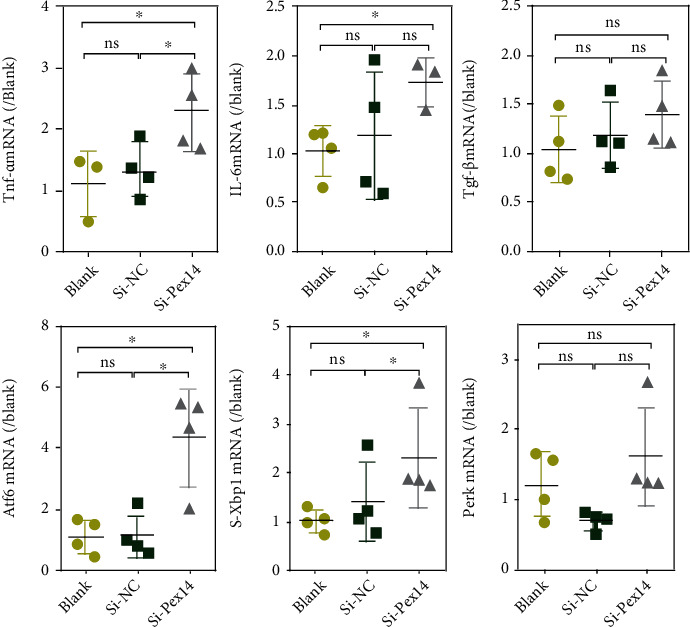
Effects of Pex14 suppression on proinflammation factors and regulators of ERS. The three proinflammation factors and three ERS regulators were detected by q-PCR. ^∗^*p* < 0.05, vs. the blank or si-NC group.

ROS play a critical role in the signaling cascade of inflammatory factors and endoplasmic reticulum stress (ERS). Therefore, we examined the OS/inflammatory response in Pex14 KD cells. As shown in Figure 4(a), Tnf-*α* and IL-6 mRNA level was significantly enhanced in Pex14 KD cultures. Atf6 and spliced-Xbp1 mRNA levels were also increased (Figure 4(b)). These observations indicate that OS caused by deletion of Pex14 leads to the upregulation of the proinflammatory factors and activates ERS.

### 3.4. Effect of Pex14 KD on INS-1 Cell Viability after Exposure to FA

**Figure 5:**
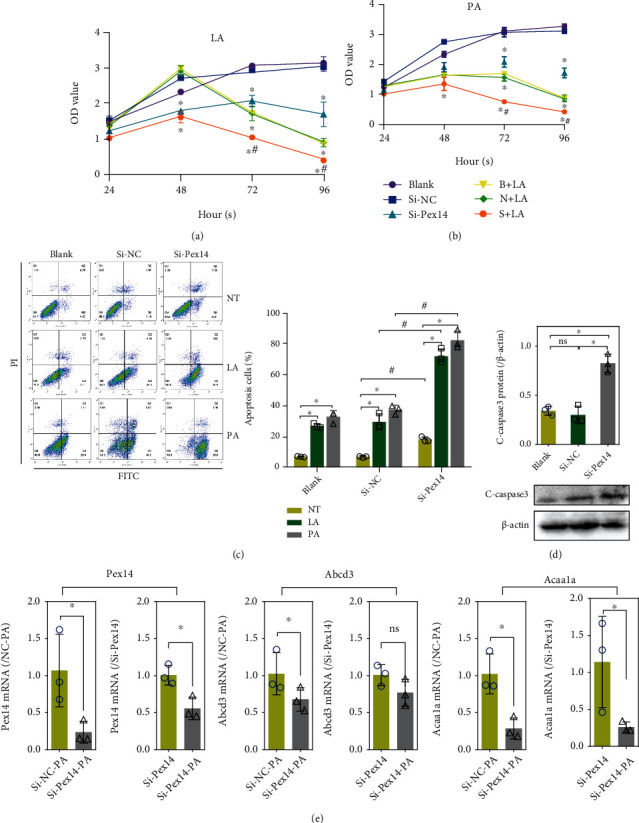
Knockdown of Pex14 aggravated fatty acid-induced apoptosis. (a, b) Cell Counting kit-8 assay was used to evaluate the effect of Pex14 KD on INS-1 cell viability with or without LA (a) or PA (b) treatment. ^∗^*p* < 0.05, vs. the time-matched blank or si-NC group. ^#^*p* < 0.05, vs. the si-NC with or without LA/PA group. (c) Cell apoptosis was assessed by flow cytometry with an Annexin V-PI kit. ^∗^*p* < 0.05, vs. the NT or LA group. ^#^*p* < 0.05, vs. the si-NC with or without LA/PA group. (d) The apoptosis regulators c-caspase3 were detected by immunoblot assay. (e) q-PCR detect the transcription of Pex14, Abcd3, and Acaa1a in Pex14 KD cells with PA treatment. ^∗^*p* < 0.05, vs. the blank or si-NC group.

A CCK8 assay showed that Pex14 KD significantly reduced INS-1 cell viability. This tendency was further strengthened by LA or PA exposure (Figures 5(a) and 5(b)). A dramatic reduction in cell viability was observed after 48 h in blank cells treated with PA, whereas in blank cells exposed to LA, a significant decrease in cell viability was not observed until 72 h. This indicates that the lipotoxicity of PA is greater than that of LA, which is consistent with a previous report [10].

### 3.5. Effects of Pex14 KD on INS-1 Cell Apoptosis and Autophagy

We examined the impact of Pex14 KD on programmed cell death including apoptosis and autophagy in cells treated with FAs. Pex14 KD caused a slight increase in the number of early apoptosis cells (Q2) and late apoptosis or necrotic cells (Q4) (Figure 5(c)). The rates of cell apoptosis and necrosis were dramatically increased in Pex14 KD cells exposed to LA or PA; especially in PA exposure, the number of apoptotic and necrotic cells increased to 90%. Pex14 KD upregulated the protein expression of the proapoptotic factors cleaved-caspase 3 (Figure 5(d)). The results of the apoptosis assay were consistent with the cell viability assay data, and both indicated that Pex14 KD exacerbated the FA-induced lipotoxicity of *β* cells, especially in response to PA exposure. Consistently, we showed that PA exposure could further reduce Pex14, Abcd3, and Acaa1a expression in INS-1 cells (Figure 5(e)). Moreover, the fact that PA induced more apoptotic cell death in Pex14 KD cells than LA could explain the decrease in detectable ROS levels in PA-exposed cells.

**Figure 6:**
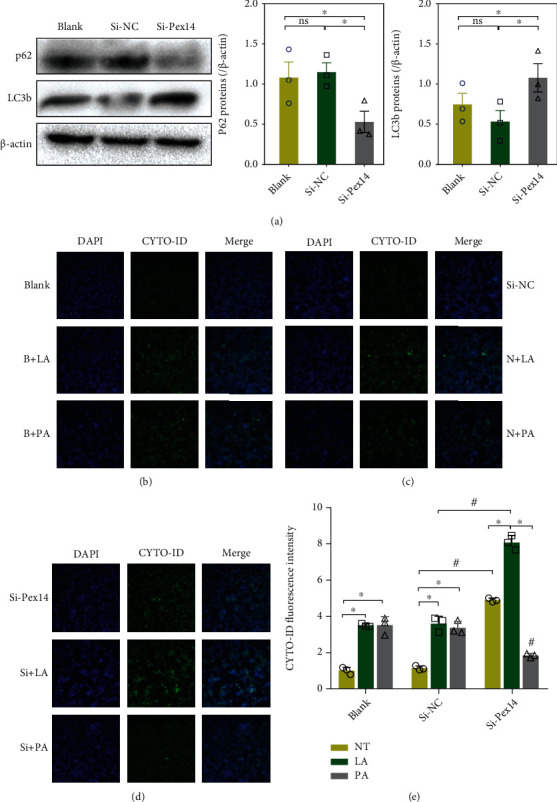
Effect of Pex14 KD on autophagy in INS-1 cells. (a) The autophagy markers were detected by western blotting; ^∗^*p* < 0.05, vs. the blank or si-NC. (b–e) Autophagy vacuoles were detected by flow cytometry with Cyto-ID fluorescence dye in blank cells (b), si-NC group (c), and si-Pex14 cells (d) with LA or PA treatment. ^∗^*p* < 0.05, vs. the NT or LA group. ^#^*p* < 0.05, vs. the si-NC with or without LA/PA group.

Next, we examined the effect of Pex14 KD on autophagy. Increased LC3b and decreased p62 expressions indicated the activation of autophagy in Pex14 KD cells (Figure 6(a)). Pex14 KD increased the number of autophagy vacuoles detected using the CYTO-ID autophagy detection kit. Moreover, autophagy vacuoles were markedly increased upon treatment of Pex14 KD cells with LA. PA exposure also led to a moderate increase in autophagy vacuoles compared with the blank groups. However, in Pex14 KD cells, the number of detectable autophagy vacuoles decreased after PA exposure (Figures 6(b)–6(e)). These results indicate that Pex14 KD can lead to the simultaneous activation of apoptotic and autophagic cell deaths.

### 3.6. Pex14 KD Impairs Insulin Secretion

**Figure 7:**
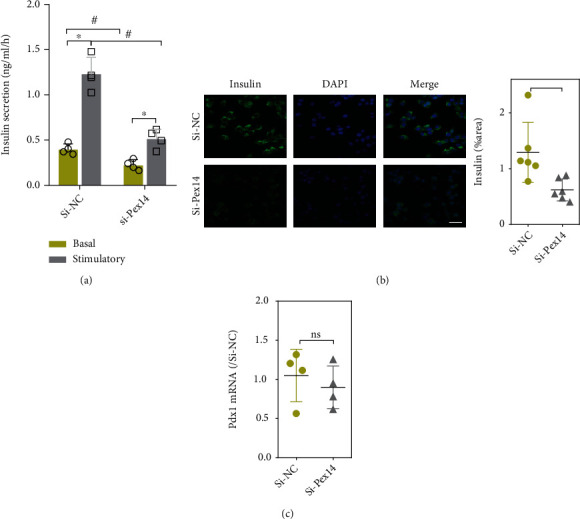
Effect of Pex14 KD on INS-1 cells function. (a) Analysis of insulin secretion in INS-1 cells with Pex14 KD. ^∗^*p* < 0.05, vs. basal. ^#^*p* < 0.05, vs. the si-NC with or without stimulatory. (b) The mRNA level of transcription factors PDX1 was detected by q-PCR. (c) Insulin expression was detected by IF staining. ^∗^*p* < 0.05, vs. the si-NC group.

We examined glucose-stimulated insulin secretion in Pex14 KD INS-1 cell. ELISA assay revealed that Pex14 KD caused blunted glucose-stimulated insulin secretion in INS-1 cell. IF staining also showed reduced insulin in Pex14 KD cells (Figures 7(a) and 7(b)). To explore the mechanism underlying impaired insulin secretion, we examined the expression of the PDX1 gene that regulates insulin secretion. However, Pex14 KD showed no effect on the mRNA level of Pdx1 (Figure 7(c)).

## 4. Discussion

IUGR increases the susceptibility to T2D and impairs glucose tolerance in adults [12, 13]. We previously reported that maternal protein malnutrition leads to aberrant expression of Pex14 and FA metabolic enzymes in the fetal pancreas using an IUGR rat model [14]. Our knockdown assays using Pex14 siRNA support the hypothesis that downregulation of Pex14 lead to programed cell death and reduced resistance to lipotoxicity in the *β* cell.

Cells regulate the number of peroxisomes through a dynamic interplay between biogenesis and degradation. Pex14, a component of the docking complex on the peroxisomal membrane, functions in the dual processes of peroxisomal biogenesis and degradation in response to environmental changes [17]. Here, we showed that Pex14 KD downregulated the expression of Pex3, Pex5, and Pex19, all functioning in membrane protein insertion, inducing de novo biogenesis of peroxisomes. Previous reports show that depletion of Pex3 or Pex9 leads to complete loss of peroxisomal function [16, 18, 19]. Pex11b, a cellular protein that induces peroxisome division, [20] was also downregulated upon Pex14 KD. Pex11b gene mutations may cause peroxisome biosynthesis disorders [21, 22], and its overexpression results in increased numbers of peroxisomes [23]. Pex14 KD also upregulated Pex5, a cytosolic receptor for matrix protein docking at Pex14 [24]. Of note, downregulation of the transcription factor Ppar-*γ* and Pgc-1*α*, both regulating peroxisome proliferation, may have contributed to the decreased expression of the peroxins. The downregulation of peroxins indicated that defective biogenesis and division was responsible for the reduced number of peroxisomes, although the precise molecular mechanism remains to be defined.

Functional autophagic pathways play a central role in the recycling of peroxisomes. Pexophagy, the selective degradation of peroxisomes via the autophagy machinery, is related to the quantitative regulation of peroxisomes and homeostasis in the metabolism of numerous substrates including lipids [25, 26]. Pex14 directly binds to Pex5 under normal conditions and is involved in peroxisome biogenesis. It interacts with LC3-II, an essential factor for autophagosome formation, under the nutrient starved condition, and is involved in the degradation of peroxisomes [19]. Decreased Pex14 levels in HeLa cells decrease pexophagy [27]. In contrast, Pex14 KD activated autophagy in INS-1 cells. We speculate that accumulation of ROS and subsequent OS led to autophagy activation, which might be a protective response for cells.

Both saturated and unsaturated FAs such as PA and LA trigger autophagy in *β* cells, although the underlying molecular mechanisms differ [28–30]. Our results showed that LA exposure increased the number of autophagy vacuoles in INS-1 cells. Pex14 KD further markedly increased autophagy induced by LA exposure. By contrast, Pex14 KD reduced the number of detectable autophagy vacuoles in PA exposure cells. A previous study suggested that the autophagy system is activated in PA-treated cells, and the induction of autophagy might play an adaptive and protective role in PA-induced cell death [31]. The fact that PA treatment increased apoptosis in Pex14 KD cells may explain the decrease in invisible autophagy vacuoles in the PA treatment group.

Long-term exposure to FAs and their accumulation induce apoptosis in *β* cells and contribute to the pathogenesis of T2D [32]. The molecular mechanisms potentially involved in FA-induced *β* cell apoptosis include ROS production, endoplasmic reticulum stress, trigger of apoptogenic factors, and accumulation of lipid intermediate molecules [2, 3, 33]. The present study also confirmed that exposure to either the saturated or the unsaturated FA (PA/LA) induced *β* cell apoptosis. Moreover, Pex14 KD reduced INS-1 cell resistance to lipotoxicity and caused more cell apoptosis.

Pex14 KD dysregulated the key enzymes involved in peroxisomal FAO. Acox1/3 is the rate-limiting enzyme of the first step of FAO. Hsd17b4 catalyzes the second step of FAO and leads to the formation of chain-shortened acyl-CoA and acetyl-CoA [34]. Acaa1a catalyzes the final step of the peroxisomal *β*-oxidation of straight-chain acyl-CoAs [35]. Abcd3 is a peroxisomal membrane protein required for fatty acyl-CoA transport into peroxisomes. Fasn plays a critical role in the de novo synthesis of fatty acid. Pex14 KD downregulated Acox1, Hsd17b4, and Acaa1a, meanwhile it upregulated Acox3, Abcd3, and Fasn. Similar changes were observed in the IUGR pancreas. In addition, Pex14 KD decreased the expression of Cpt1a, a rate-limiting enzyme for long-chain FAO in the mitochondrion, and its deficiency results in a decreased rate of *FAO* [36]. These changes could lead to increased lipid accumulation in INS-1 cells. Given the complexity of FA metabolism, to determine the lipid profile by gas chromatography-mass spectroscopy would help to elucidate the true variation induced by peroxisome dysfunction. Recently, Baboota et al. reports that deletion of Pex5 lead to impairment of peroxisomal FAO and mitochondrial disruptions, thereby increasing *β* cell apoptosis and decreases insulin secretion in islet [37]. We speculate that downregulation of Pex14 contributes to islet dysplasia in the IUGR fetus, which still needs to be confirmed by in vivo studies.

Peroxisomes play a critical role in maintaining cellular redox homeostasis [38, 39]. Pex14 KD lead to the downregulation of catalase, which may lead to reduced degradation of H_2_O_2_. Downregulation of Sod2, a key mitochondria antioxidant catalyzing the conversion of superoxide radicals to H_2_O_2_, can also lead to an increase in superoxide radicals [40]. The present results suggest that the ROS accumulation in Pex14 KD *β* cells was caused by the absence of functional peroxisomes and subsequent mitochondrial alterations.

ROS are key signaling molecules that have important functions in regulating proinflammatory cytokines, and the two influence each other in a positive feedback loop [41–43]. Tnf-*α* and IL-6 were upregulated in Pex14 KD cells. In addition, Pex14 KD activated ER stress and proapoptotic factor in INS-1 cells. Prolonged ER stress causes pancreatic *β* cell apoptosis. Hence, induction of proinflammatory factors, ER stress, and subsequent apoptosis by the downregulation of Pex14 was a plausible explanation for our previous finding that ERS is dysregulated in the pancreas of IUGR models [43].

**Figure 8:**
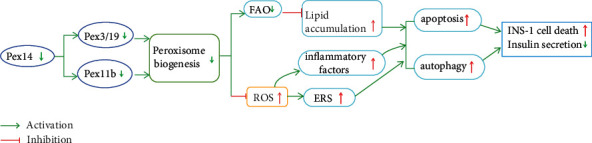
Hypothesis schematic. A unifying hypothesis depicting the cascade of events in response to Pex14 KD that result in oxidative stress and programmed cell death in *β* cells. We speculate that Pex14 KD inhibits peroxisome biogenesis, which disturbs fatty acid oxidization, thus leading to lipid accumulation. Peroxisome deficiency also causes downregulation of catalase and Sod2, in turn H_2_O_2_ accumulation. H_2_O_2_ activates inflammatory factors and ERS, which in turn induces apoptosis and autophagy.

In general, one important finding of this study was that downregulation of Pex14 plays a fundamental role in INS-1 cell viability and resistance against lipotoxicity by affecting the expression of multiple genes involved in peroxisome biogenesis, FAO, and ROS detoxification (Figure 8). However, a limit in this study is that as a rat insulinoma cell line, INS-1 cells cannot fully mimic the phenotype of primary *β* cells in vivo. And the data obtained in INS-1 cells are not fully consistent with the data obtained from investigation of the pancreas in IUGR rats, so the impact of other genetic changes on pancreatic development warrant further research. Another limit of this study is that the mechanism underlying the decrease in insulin secretion in Pex14 KD cells remains to be explored. Nevertheless, the present results suggest that downregulation of Pex14 underlies, at least in part, the pancreatic dysplasia in the IUGR fetus. Further elucidation of the role of peroxisomes in *β* cell function might contribute to improve our understanding of T2D and prevent this disease in adulthood.

## Data Availability

The data used to support the findings of this study are available from the corresponding author upon request.

## Abbreviations

Acox: Acyl-CoA oxidase
CoA: Coenzyme A
Acaa1: 3-Ketoacyl-CoA thiolase
Abcd: ABC transporter D
CAT: Catalase
Cpt1: Carnitine palmitoyltransferase-1
DCFH-DA: Dichlorodihydrofluorescein diacetate assay
FAO: Fatty acid beta-oxidation
FBS: Fetal bovine serum
Fasn: Fatty acid synthase
Hsd17b4: Peroxisomal 17*β*-hydroxysteroid dehydrogenase type 4
H_2_O_2_: Hydrogen peroxide
IF: Immunofluorescence
IUGR: Intrauterine growth restriction
Pex: Peroxin
ROS: Reactive oxygen species
Sod: Superoxide dismutase
siRNA: Small interfering RNA
T2D: Type 2 diabetes
VLCFA: Very-long-chain fatty acids.
