# Supplementary material for: Peroxisome Deficiency Dysregulates Fatty Acid Oxidization and Exacerbates Lipotoxicity in β Cells
Source: Oxid Med Cell Longev. 2021 Aug 22;2021:7726058. doi: 10.1155/2021/7726058 (PMC8405300; doi:10.1155/2021/7726058)
Supplement: Supplementary Materials — The Supplementary Material for this article can be found online. Table S1: primer sequences of q-PCR and sequence of siRNA. Table S2: antibodies for western blotting and IF. [file 7726058.f1.docx]

**Supplement table S1. Primers sequences of q- PCR and sequence of siRNA**

| **Gene name** | **Accession**  **Number** | **Primer Sequences(**5’ -3’) | **Annealing temp (℃)** |
| --- | --- | --- | --- |
| **Pex1** | NM_017364.4 | AGGCTCCCAAGACCTTACAC  TCCTCTCCACTTTGGCTCTG | 60 |
| **Pex3** | NM_003630.3 | GCACATGTATGCTGGTGGTT  GAGCTAGGATGCTAGTGCCA | 60 |
| **Pex5** | NM_001170584.1 | AGCAGCCAGTTTGTTTCCTG  AGCAGCCAGTTTGTTTCCTG | 60 |
| **Pex7** | NM_001034147.1 | ACGCCATTAGGAGGGTGAAA  TCTGTGTGATGCTCCACTGT | 60 |
| **Pex11b** | NM_001025684 | TTTAGTGCTCAGAGCCAAGC  GCTCAGATGACCCTCCAGTT | 60 |
| **Pex14** | NM_001013911 | GTACAGAGGGAGGACAGACG  TCTCGCTCACTCTCATTGCT | 60 |
| **Pex16** | XM_006234567.3 | TGCTCAATGACGGGATCCTT  TACACTCAGCCATGTCAGCA | 60 |
| **Pex19** | NM_001107375 | CAATGAAGGAGCTGGCTGAG  CTGCGTTCTGAAGGTCAGTG | 60 |
| **Abcd3** | NM_008991.2 | GTTGGGCCATATCCTTGAGC  TTGCCATCGCCATTCTTTGT | 60 |
| **Acox1** | NM_017340.2 | GAAATACGCCCAGGTGAAGC  CTGCATTTCCCACGAGGAAG | 60 |
| **Acox2** | [NM_145770](https://www.ncbi.nlm.nih.gov/nucleotide/NM_145770.2?report=genbank&log$=nuclalign&blast_rank=4&RID=DPN74MZU015) | GCCTTACACGGTGTTCTGAC  CATCCTTCCGGATCAAGGGA | 60 |
| **Acox3** | [NM_053339](https://www.ncbi.nlm.nih.gov/nucleotide/NM_053339.1?report=genbank&log$=nuclalign&blast_rank=24&RID=DPN9WC8S01R) | CTAGCCATGAACCGGTTTGG  TGTGAAGTGAGCTCCGTCTT | 60 |
| **Hsd17b4** | NM_024392 | ATGCCAGAAGACCTCGTTGA  TGCTCCAACCTCAAACAAGC | 60 |
| **Hsd17b13** | [NM_001009684](javascript:if(window.name=='')%20%7b%20window.location.href='./nil';%20%7d%20else%20%7b%20dynPopitupType('NCBI_REFSEQ__TRANSCRIPT',%20'http://www.ncbi.nlm.nih.gov/entrez/query.fcgi?db=nucleotide&term=NM_001009684');%20%7d) | AGGAGCTGGACACGGAATAG  TCCTCAACACCGTGCTTACT | 60 |
| **Mfe1** | NM_133606.1 | GCTATCGAGTGACTGAGCCT  TGGATATCAGCACCTGCACA | 60 |
| **Acaa1a** | NM_012489.2 | GCCCAATTTCTGAGTGGCAT  TCCCGATGAACACTGTCTGT | 60 |
| **Cpt1a** | NM_001876.4 | CTGGGCATGATCGCAAAGAT  AGCCCTGTACCAAAGAGGAC | 60 |
| **Prdx5** | NM_053610.1 | GATCAAGGTGGGAGACACCA  AACAGCCAGGTGTAAATGCC | 60 |
| **Tnf-α** | NM_012675.3 | CTCAGATCATCTTCTCAAAA  AGGTACAGCCCATCTGCTGGT | 60 |
| **IL-6** | NM_012589.2 | CCACTGCCTTCCCTACTTCA  TTGCCATTGCACAACTCTTT | 60 |
| **Tgf-β** | NM_021578.2 | GACATGAACCGACCCTTCCT  TTCTCTGTGGAGCTGAAGCA | 60 |
| **Atf6** | NM_001107196.1 | GATTTGATGCCTTGGGAGTC  GGACCGAGGAGAAGAGACAG | 60 |
| **S-Xbp-1** | NM_001004210.2 | AAACAGAGTAGCAGCACAGACTGC  TCCTTCTGGGTAGACCTCTGGGAG | 60 |
| **Perk** | NM_031599.2 | GTGCTCCGCTTATTCCTTT  CCTGTCTTGGTTGGGTCTG | 60 |
| **β-actin** | NM_031144 | AGTCCCTCACCCTCCCAAAAG  AAGCAATGCTGTCACCTTCCC | 60 |
| **Si-Pex14** |  | GCAGAAGGUCCAGGAGCUATT  UAGCUCCUGGACCUUCUGCTT |  |
| **Si-NC** |  | UUCUCCGAACGUGUCACGUTT  ACGUGACACGUUCGGAGAATT |  |

**Supplement table S2. antibodies for western blotting and IF**

| **Target**  **protein** | **MW (kDa)** | **Dilution** | **Company** | **Ref.** |
| --- | --- | --- | --- | --- |
| Pex14 | 57 | 1:1000 | Proteintech | 105941-1-lg |
| Pex3 | 37 | 1:1000 | Proteintech | 10946-1-AP |
| Pex11b | 28 | 1:1000 | Immunoway | YT3675 |
| Pex19 | 37 | 1:1000 | Enogene | E2A7361 |
| Abcd3 | 74 | 1:1000 | Proteintech | 10365-1-AP |
| Hsd4-17β | 48 | 1:500 | Immunoway | YT5386 |
| Cpt1a | 85 | 1:1000 | Immunoway | YN3388 |
| Ppara | 50 | 1:2000 | Enogene | E1A5301 |
| Pgc-1α | 91 | 1:300 | Enogene | 66369-1-Ig |
| Sod2 | 25 | 1:2000 | Abbclonal | A19576 |
| Sod3 | 26 | 1:500 | Affinity | DF7753 |
| Catalase | 60 | 1:400 | Proteintech | 66765-1-AP |
| LC3b | 15 | 1:1000 | CST | 2775 |
| P62 | 62 | 1:1000 | Abclonal | A7758 |
| Caspase 3 | 17 | 1:1000 | Proteintech | 19677-1-AP |
| β-actin | 43 | 1:3000 | Proteintech | 60008-1-lg |
